# Dynamic Modeling and Performance Analysis of a Hip Rehabilitation Robot

**DOI:** 10.3390/biomimetics8080585

**Published:** 2023-12-03

**Authors:** Zengyu Jia, Ruiqin Li, Juan Liu, Yuan Wang

**Affiliations:** School of Mechanical Engineering, North University of China, Taiyuan 030051, China; b20210204@st.nuc.edu.cn (Z.J.);

**Keywords:** hip joint rehabilitation robot, dynamic modeling, Newton–Euler method, dynamic performance index

## Abstract

The dynamic performance of a 2-DOF hip joint rehabilitation robot configuration for patients with hip joint dyskinesia was analyzed. There were eight revolute pairs on one side of the hip joint rehabilitation robot configuration. The dynamics of the robot configuration were analyzed with the Newton–Euler method, and a dynamic model was developed. On the basis of the solved dynamic model, the dynamic performance index of the hip joint rehabilitation robot configuration is given, and the performance atlas under different parameters is drawn. The performance of the hip joint rehabilitation robot is theoretically verified. This study provides a theoretical basis for the research and development of exoskeleton rehabilitation robots.

## 1. Introduction

As society ages, the number of people with lower limb dyskinesia is increasing rapidly [1,2]. This is leading to an increase in research in the field of rehabilitation robots [3,4]. The hip joint [5] can be regarded as the largest and most stable joint in the body, and it is an important joint as it is subjected to the most stress.

The emergence of hip exoskeleton robots [6] is good news for patients affected by hip dyskinesia, but most exoskeleton rehabilitation robots are tandem mechanisms, such as Alex [7] and LOPES [8], meaning that they are characterized by a poor dynamic performance and poor structural stability. People have also considered the use of a parallel mechanism to improve the performance of rehabilitation robots which has some significant kinematic and dynamic characteristics [9]. Guzman-Valdivia et al. [10] designed a hip joint rehabilitation robot with 5-DOFs for combinatorial motion. This simple mechanism can meet the stability requirements for hip joint rehabilitation. Hsu et al. [11] designed a hip exoskeleton, which is a kinematically compatible four-degree-of-freedom robot. According to the effect of dynamic assistance, it was verified that the exoskeleton robot can provide a stable auxiliary torque for the hip joint and help the patient carry out effective rehabilitation training.

Zhang et al. [12] proposed two configurations, namely 3-UPS/S and 2-RPS/UPS/S, that, when combined with the human hip joint, are metaplastic parallel mechanisms suitable for lower limb rehabilitation. The two configurations can be converted into each other so as to meet the needs of patients at different stages of rehabilitation, and they have good motion characteristics. All of these examples illustrate the advantages of a parallel mechanism applied in the field of rehabilitation. Dynamic modeling/analysis [13] of rehabilitation robots based on a parallel mechanism [14,15,16], so as to obtain the dynamic performance index of the mechanism, plays an important role in establishing the feasibility of the parallel robot theory. Hou et al. [17] established the dynamic model of a 3RSR parallel mechanism using the Newton–Euler method and determined the influencing factors that affected the mechanism performance. Sokolov et al. [18] carried out a dynamic analysis of an R-P-S parallel mechanism with 3-DOFs using the virtual work principle and obtained the equation of motion in the coordinate system of the end-effector. Through calculation and program comparison, the feasibility of this mechanism was proved. Li et al. [19] proposed a 5PSS/UPU parallel mechanism. Because the connecting link leads to elastic deformation, a method that combines the finite element and Lagrange methods is used to simulate and verify the mechanism dynamics, and the results show that this method is feasible and the mechanism demonstrates good performance. Both the series mechanism and parallel mechanism have their own advantages. The series mechanism consists mainly of an open-loop structure, its workspace is relatively large, its load is relatively small, and the speed is fast. The parallel mechanism [20,21] has a closed-loop structure with a large load, relatively high precision, and relatively slow speed. Currently, the parallel mechanism is widely used in the field of exoskeleton robots [22,23,24].

The authors presented a symmetric 2-DOF hip joint rehabilitation robot configuration in a previous study [23] which was composed of two branch chains in parallel. The mechanism can realize the anterior flexion/extension and abduction/adduction movement of the hip joint. Differences between this mechanism and other mechanisms include the use of a spatial linkage mechanism, the presence of fewer components, the fact that less drive is required, the simple structure, and the fact that its usage is typically associated with a lighter device; in terms of function, it has certain advantages over the current lower limb rehabilitation robot, overcoming some of its shortcomings.

Based on a kinematic performance analysis, this paper further analyzes the dynamic performance of a hip joint rehabilitation robot. This paper is organized as follows: in Section 2, the hip joint rehabilitation robot configuration and its joint arrangement are introduced. In Section 3, the velocity and acceleration of the hip joint rehabilitation robot configuration are analyzed, and the velocity and acceleration curves of the corresponding motion types are given. Then, in Section 4, the dynamic modeling of the mechanism is conducted using the Newton–Euler method and the dynamic performance index of the robot is given. Finally, conclusions are drawn in Section 5.

## 2. Configuration and Joint Distribution of Hip Joint Rehabilitation Robot

**Figure 1:**
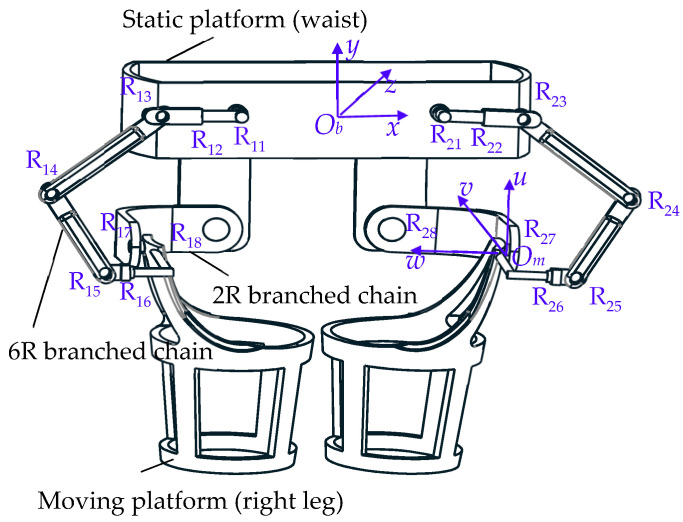
Model of robot and coordinate system.

The hip joint rehabilitation robot proposed in this paper, because of its spatial 8R structure, cleverly uses the idea of connecting six rotating pairs in a series and then connecting two rotating pairs in parallel, making it novel and original. The configuration of the 2-DOF hip joint rehabilitation robot is shown in Figure 1. A single side is formed by a static platform, a moving platform, a 6R branch chain, and a 2R branch chain in parallel (due to its left and right symmetry, no more statements will be made). The static platform is the waist exoskeleton of the robot, and the moving platform is the thigh exoskeleton. The distribution positions of the eight kinematic pairs of the robot are shown in Figure 1, where the static coordinate system of *O_b_*-*xyz*, *O_m_*-*uvw* represents its moving coordinate system, the *y*-axis is parallel to the axis of the rotating pair R_11_ and R_18_, the axis of R_12_ and R_16_ is parallel to the *x*-axis, and the axis positions of the other kinematic pairs are obvious and will not be further described.

The midpoint of R_11_ and R_21_ is set as the origin *O_b_* of the static coordinate system, the *x*-axis direction coincides with the connection direction of *O_b_*R_21_, the *y*-axis direction is perpendicular to the R_11_R_21_ connection dimension, and the *z*-axis direction is perpendicular to the upward direction. The center of rotation of the rotating pair R_27_ is set as the origin *O_m_* of the moving coordinate system, the *u*-axis is vertical and in an upward direction along the R_27_-axis, the *v*-axis is vertical and in an inward direction along the R_27_-axis, and the *w*-axis and the R_27_-axis merge to the left. The branch chain coordinate system R_1*i*_-*x*_1*i*_*y*_1*i*_*z*_1*i*_ (*i* = 1.8) is established at the rotation center of the revolute pair R_1*i*_. The *x*-axis direction of the branch chain coordinate system R_11_-*x*_11_*y*_11_*z*_11_ is along the R_11_-axis, the *y*_11_-axis is to the left along the R_11_R_12_ line direction, and the *z*_11_-axis is in a vertically downward direction.

## 3. Velocity and Acceleration Analysis of the Hip Joint Rehabilitation Robot Configuration

In order to calculate the inertial force of each link in the branch chain, it is necessary to analyze the velocity and acceleration of the branch chain first. The velocity and acceleration of the 6R branch chain and 2R branch chain of the robot configuration in the workspace and the velocity and acceleration of their joint space can be described by the coordinate transformation matrix, whose element is the expression of the variable of the branch chain joint.

As the hip joint rehabilitation robot configuration has 2-DOFs, and its drivers are set to R_21_ and R_27_ according to the required movement type, it can be seen from the positions of the drivers that the five links mainly drive the mechanism to perform the abduction and adduction movement. When analyzing the 6R branch chain, the kinematic pair R_22_ can be fixed, and the five links can be simplified into four links for analysis. At this time, the 6R branch chain becomes a 5R branch chain, which does not affect the kinematics of the mechanism. In the subsequent analysis of the 6R branch chain, the analysis was carried out with four links. The 2R branch chain is equivalent to a link, and the number of active links on one side of the overall symmetric mechanism is five; the serial number of a link equivalent to the 2R branch chain is denoted as l_7_, and its joint variables are denoted as θ_7_ and θ_8_.

Through the improved D-H parameters, the coordinate transformation matrix [25] between the coordinate systems of links is established, and the general formula of the transformation matrix is

(1) Tii−1=cθi−sθi0ai−1sθicαi−1cθicαi−1−sαi−1−disαi−1sθisαi−1cθisαi−1cαi−1dicαi−10001

where α_*i*−1_ is the rotation degree from *z_i_*_−1_ to *z_i_* along the *x_i_*_−1_-axis; *a_i_*_−1_ is the length from *z_i_*_−1_ to *z_i_* along the *x_i_*_−1_-axis; and *d_i_* is the length from *x_i_*_−1_ to *x_i_* along the *z_i_*-axis.

Table 1 and Table 2 show the D-H parameters of the two branch chains.

The translation vector of each link relative to the previous coordinate system, that is, the length of the link, can be obtained from Equation (1) The 5R branch chain is:

(2) $$\begin{array}{l} \left\{\begin{array}{l} {}^{i}p_{{}_{i+1}}^{5}={\left(l_{i}\;0\;0\right)}^{Τ}\;\;i=0,1,2,3\\ {}^{i}p_{{}_{i+1}}^{5}={\left(0\;-l_{i}\;0\right)}^{Τ}\;\;i=4,5 \end{array}\right.\\ \;l_{0}=0 \end{array}$$

The 2R branch chain is:

(3) p120=0 0 0Τp221=l7 0 0Τp322=l8 0 0Τ

In Formulas (2) and (3), superscripts 5 and 2 in the upper right corners represent each coordinate system of the 5R branch chain and 2R branch chain, respectively; *l*_5_ and *l*_8_ represent the translation vector from the last joint of each branch chain to the moving platform.

The link is a homogeneous round link. In the following analysis, the centroid of each link is represented in its link coordinate system as follows:

(4) $$\begin{array}{l} {}^{i+1}p_{c_{i+1}}^{5}=\frac{{}^{i+1}p_{i+2}^{5}}{2}\;i=0,1,2,3\\ {}^{i+1}p_{c_{i+1}}^{2}=\frac{{}^{i+1}p_{i+2}^{2}}{2}\;i=0,1 \end{array}$$

Before establishing the dynamic equation of each link, the velocity and acceleration of each link should be determined first. When describing the motion of each link of the two branch chains, the base coordinate system {0} is taken as the reference coordinate system, and *v_i_* and *ω_i_* represent the linear velocity and angular velocity, respectively, of the link coordinate system {*i*} relative to the reference coordinate system {0}. The linear and angular velocities of link *i* are *^i^v_i_* and^*i*^*ω_i_*, respectively, and these vectors are represented in the coordinate system {*i*}*. ^i^*^+1^*v_i_*_+1_ and^*i*+1^*ω_i_*_+1_ represent the linear velocity and angular velocity of the link coordinate system {*i*+1}, respectively. A glossary of the symbols involved is shown in Table 3.

Based on this, the recursive formula of link velocity and acceleration can be obtained.

The angular velocity recurrence formula for a link is as follows:

(5) ωi+1i+1=Rii+1ωii+θ˙i+1zi+1i+1

The linear velocity recurrence formula of a link is:

(6) νi+1i+1=Rii+1νii+ωii×pi+1i

The recursive formula of angular acceleration of a link is:

(7) ω˙i+1i+1=Rii+1ω˙ii+zi+1i+1θ¨i+1+Rii+1ωii×zi+1i+1θ˙i+1

The linear acceleration recursive formula of a link is:

(8) ν˙i+1i+1=Rii+1ν˙ii+ω˙ii×pi+1i+ωii×ωii×pi+1i

Acceleration of the centroid of the link is:

(9) ν˙ci+1i+1=ν˙i+1i+1+ω˙i+1i+1×pci+1i+1+ωi+1i+1×ωi+1i+1×pci+1i+1

In this situation, $\dot{\theta }$*_i_*_+1_ is the angular velocity of the joint, *^i^*^+1^*z_i_*_+1_ is the *z*-axis unit vector of {*i*+1}, and the subscript c is the center of mass of each link.

zi+1i+1=0 0 1Τ

Rii+1=cosθi+1sinθi+10−sinθi+1cosθi+10001

Because the static platform is stationary, the following formula is obtained:

(10) ω050=v050=ω˙050=0ω020=v020=ω˙020=0

Considering that the mechanism is affected by gravity, this formula is obtained:

(11) v˙050=g50=0 g 0Τv˙222=g20=0 g 0Τ

According to Equations (5) and (6), the velocity of each link of the branch chain relative to the base coordinate system {0} is:

(12) ωi+1=Ri+10Τωi+1i+1vi+1=Ri+10Τvi+1i+1

According to Equations (7) and (8), the acceleration of each link relative to the base coordinate system {0} is:

(13) ω˙i+1=Ri+10Τωi+1i+1v˙i+1=Ri+10Τvi+1i+1

where the transformation matrix of the 2R branch chain, represented by 6 in its {0} coordinate system, is still substituted into the general formula of the transformation matrix, so that the transformation matrix R89 is obtained.

Ri+10Τrepresented∏i=0i+1Rii+1Τ i=0∼4,6,7

(14) R56=R89=1 0 00 1 00 0 1

R89 is found by rotating the matrix ***R***, which is the identity matrix. Equations (12) and (13) refer to the velocity and acceleration of each link of the two branch chains. The next step of dynamic modeling can be carried out based on the velocity and acceleration obtained.

Based on the established velocity and acceleration model, the velocity and acceleration of the robot were simulated [26] in forward flexion, extension, abduction, and adduction. The results are as follows.

**Figure 2:**
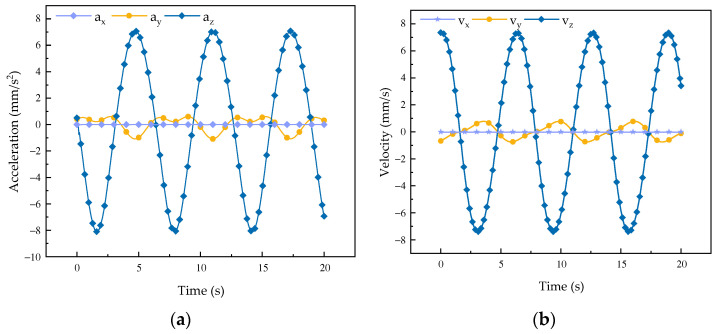
Changes to the robot flexion and extension curves. (**a**) Velocity curve; (**b**) acceleration curve.

**Figure 3:**
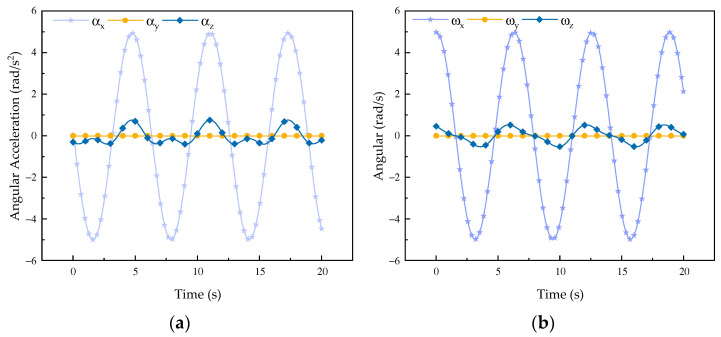
Curves of forward flexion and extension motions of the robot. (**a**) Angular velocity curve*;* (**b**) angular acceleration change curve.

It can be observed from the change curves of velocity and acceleration in Figure 2 and Figure 3 that during forward flexion and extension of the rehabilitation robot configuration its velocity and acceleration change most obviously along the *x*-axis, while there is little change along the *y*-axis and no change in velocity along the *z*-axis. According to its movement direction, it can be concluded that the change curve is a regular sine curve, which is in accordance with the motion characteristics of the robot during forward flexion and extension motions. It can be seen from the curve of angular velocity and angular acceleration that the motion curve of the mechanism around the *x*-axis and the *y*-axis changes in an obvious manner, while the motion curve around the *z*-axis does not change, so the curve is always 0, which is determined by the overall characteristics of the robot. From the simulation curve of velocity and acceleration of forward flexion and extension motions, it can be concluded that the mechanism shows good performance.

**Figure 4:**
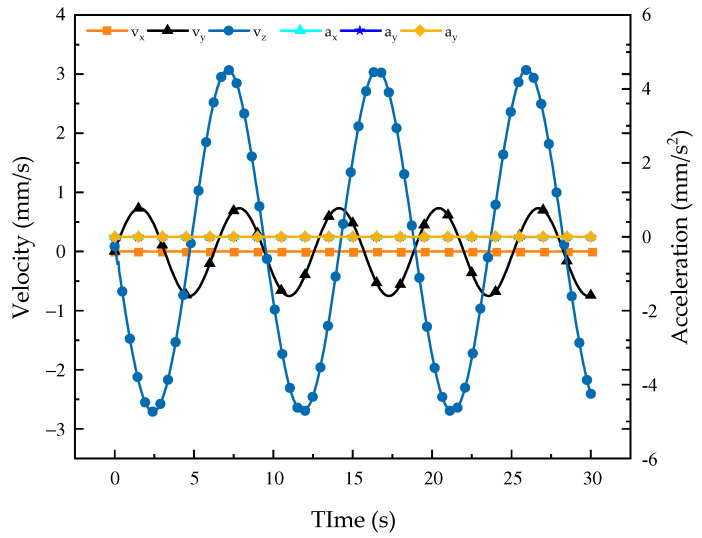
Velocity and acceleration curves of mechanism abduction and adduction motions.

**Figure 5:**
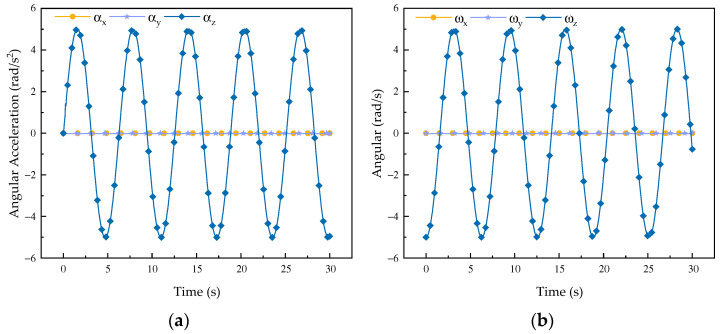
Change curves of the robot abduction and adduction motions. (**a**) Angular velocity curve*;* (**b**) angular acceleration variation curve.

According to the change curves of velocity and acceleration in Figure 4 and Figure 5, during the abduction and adduction movements of the robot configuration, the main change in its velocity is along the *x*- and *y*-axes, while there is no change in velocity along the *z*-axis and the change of acceleration is 0; additionally, its curve presents a regular sinusoidal change, indicating that the mechanism is stable and controllable during the abduction and adduction movements. From the curve of angular velocity and angular acceleration, it can be observed that the motion curve of the mechanism around the *z*-axis changes in an obvious manner, while it does not change around the *x*- and *y*-axes. It is obvious that the mechanism is undergoing stable and smooth abductive and adductive motions, which proves the feasibility of the mechanism design. Through analysis, the velocity and acceleration of the robot are obtained and simulated. According to the simulation, the kinematic performance of the robot is relatively good, which lays the foundation for the next step of dynamic modeling.

## 4. Dynamic Modeling and Performance Analysis

### 4.1. Establishment of Dynamic Equation

The Newton–Euler method [25,27,28] was used to model the dynamics of the robot configuration. Compared to the Lagrange method, the Newton–Euler method [29,30] is more suitable for low-velocity moving systems, as its physical meaning is clearer and its computational efficiency is higher. Newton’s second law and Euler’s equation are the basis for the establishment of the Newton–Euler dynamics model. Newton’s second law describes the dynamic characteristics of the link moving with the center of mass, and Euler’s equation describes the dynamic characteristics [31] of the link rotating around the center of mass.

The force balance equation of the link *i* is:

(15) Fii−Ri+1iFi+1i+1−migi=miv˙ci

*^i^**F**_i_* is the force acting on link *i* by link *i*-1;

Ri+1i is the transpose of Rii+1;

*m_i_* is the mass of link *i*;

*m_i_**v***_c*i*_ is the inertial force of link *i*.

As the gravity on the link was already considered when determining velocity and acceleration, it is not taken into account in the establishment of the force balance equation. Based on Equation (15), the force balance equations of each joint of the two branch chains can be written as follows:

(16) F151−R21F252=m1v˙c1F252−R32F353=m2v˙c2F353−R43F454=m3v˙c3F454−R54F555=m4v˙c4F555−R65F656=m5v˙c5

(17) F211−R87F222=m7v˙c7F222−R98F323=m5v˙c8

where ^1^***F***_1_ is the force exerted by the outside world (including the base and the driver) on the driving links R_11_ and R_81_, and ^5^***F***^5^_5_ and ^2^***F***^2^_2_ represent the force of the 5R branch chain and 2R branch chain on the moving platform through the revolute pairs R_15_ and R_18_, respectively. Both ^6^***F***^5^_6_ and ^3^***F***^2^_3_ are 0.

The inertial force and inertial moment need to be considered in the process of movement. For link *i*, its inertial force and inertial moment can be expressed as:

(18) Fcii=mivci

(19) τcii=Iciiω˙ii+ωii×Iciiωiii

Since each link of the two branch chains is equivalent to a homogeneous round link, Icii in Equation (19) is:

Icii=12miri2000mi12li+3ri2000mi12li+3ri2

where *r_i_* is the radius of the homogeneous link.

Being the same force equilibrium equation for link *i*, the torque equilibrium equation in its own coordinate system is:

(20) τii−τcii−Ri+1iτi+1i+1−pi+1i×Ri+1iFi+1i+pcii×Fcii−pcii×τigi=0

The torque balance equations of each joint of the two branch chains can be obtained from Equation (20) as follows:

(21) τ151=τc151+R221τ25+p251×R121F25+pc151×Fc151τ252=τc252+R332τ35+p352×R232F35+pc252×Fc252τ353=τc353+R443τ45+p453×R343F45+pc353×Fc353τ454=τc454+R554τ55+p554×R454F55+pc454×Fc454τ555=τc554+R665τ65+p655×R565F65+pc555×Fc555

(22) τ121=τc121+R287τ22+p221×R187F22+pc121×Fc121τ222=τc222+R398τ32+p322×R298F32+pc222×Fc222

In Equations (21) and (22), both τ656 and τ323 are 0.

Through the above solution, the required torque of each joint can be obtained. The torque component of the connecting link interacting on the *z*-axis is as follows:

(23) τi=τiΤizii

According to Equation (23),

(24) $$\tau =\left(\begin{array}{l} \tau_{1}^{5}\\ \tau_{2}^{5}\\ \tau_{3}^{5}\\ \tau_{4}^{5}\\ \tau_{5}^{5}\\ \tau_{1}^{2}\\ \tau_{2}^{2} \end{array}\right)$$

This is the dynamic equation of the robot configuration.

### 4.2. Initial Simulations

**Figure 6:**
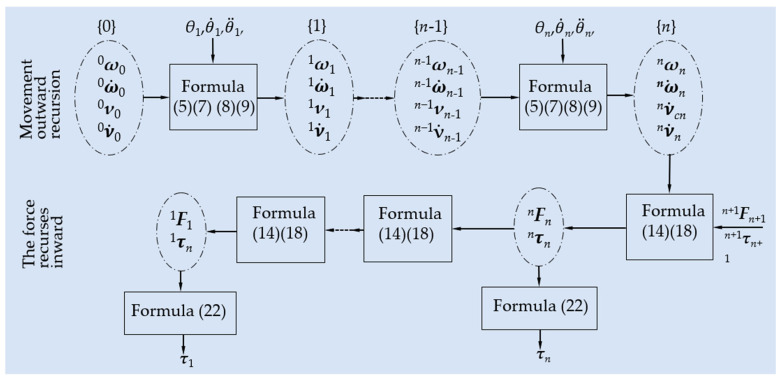
Flow chart of Newton–Euler dynamics algorithm.

The structure of the Newton–Euler dynamic algorithm is shown in Figure 6.

Based on the dynamic equation, the force and torque of the end-effector (moving platform) of the robot were simulated by the forward flexion, backward extension, and abductive adduction motions. The results are as follows.

**Figure 7:**
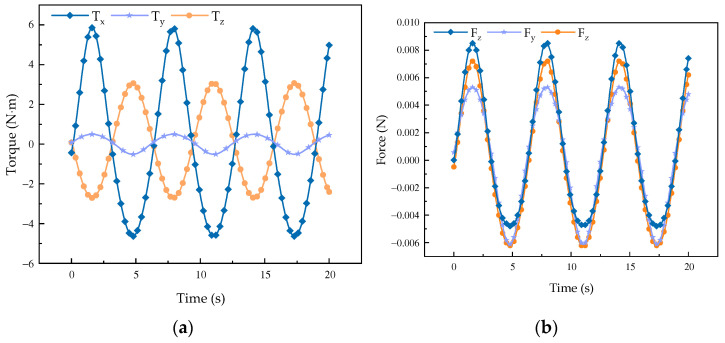
Changing curves of kinematic force and moment of the mechanism in flexion and extension. (**a**) Curve of force change; (**b**) curve of moment change.

As shown in Figure 7, the external force on the mechanism changes little during the forward flexion and extension movements, and the force change curve is obtained as shown in Figure 7a. The force change is more regular and there is no obvious mutation point. Each axis is subjected to torque action, and, as it mainly moves around the *x*-axis, the change in the torque around the *x*-axis is the most obvious. The change in the torque around the other two axes is also relatively stable with no obvious fluctuations, a phenomenon which theoretically indicates that the mechanism has good performance.

**Figure 8:**
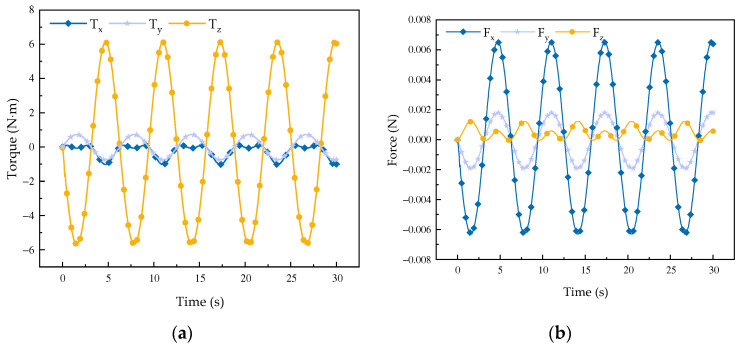
Variation curve of receiving force and moment in mechanism abduction. (**a**) Curve of force change; (**b**) curve of moment change.

As it can be observed in Figure 8, during the process of the mechanism moving abductively and adductively, the external force in the *x*-direction xperiences the most obvious change, while relatively small changes occur in the *y*-axis and *z*-axis. According to the data in the figure, the whole mechanism is less affected by external force, and the stress change curve is shown in Figure 8a. The three axes are all affected by torque. Since the robot mainly moves around the *z*-axis, this axis is mainly affected by torque, and the torque engendered by the robot abduction and adduction motions also occurs around the *z*-axis. The change in the torque around the other two axes is not very obvious, and the curve is smooth without a sudden breakpoint.

It can be found through the simulation curve of the force and torque of the robot that, in the process of forward flexion, extension, and abductor adduction, the mechanism moves smoothly and is not affected by the external factors that obviously affect the mechanism performance, a fact which proves that the mechanism can be applied to hip rehabilitation movements.

### 4.3. Dynamic Performance Index

A correct evaluation of the robot dynamic performance [32,33] plays an important role in the robot mechanism design, workspace selection, and control scheme formulation. Based on the dynamic equation, a dynamic operable ellipsoid (DME) is used as the dynamic performance index of the hip joint rehabilitation robot configuration [34]. Compared with dynamic performance indexes such as joint torque, and the mass of the connecting links and the inertia tensor, the dynamic manipulability ellipsoid [35] has the advantages of comprehensiveness, intuitiveness, universality, and high computational efficiency. The dynamic manipulability ellipsoid can describe both the linear motion and the rotational motion of the mechanism, while the joint torque, the mass of the connecting links, and the inertia tensor can only describe some of the dynamic characteristics of the mechanism. The dynamic manipulability ellipsoid can directly represent the motion range and motion constraints of the mechanism, can be applied to various types of mechanisms, and the calculation efficiency is relatively high. Taking it as a dynamic performance index can provide a more comprehensive, intuitive, universal, and effective optimization method and tool for the development and design of rehabilitation robot mechanisms.

Formula (24) can be written as follows:

(25) $$\tau =D\left(\theta \right)\ddot{\theta }+h\left(\theta ,\dot{\theta }\right)+G\left(\theta \right)$$

where ***D***(*θ*) is the mass matrix, ***h***(*θ*, $\dot{\theta }$) is the centrifugal force and Coriolis force vector, and ***G***(*θ*) is the gravity vector.

After rearranging Equation (25), the acceleration expression of the mobilization platform can be written as follows:

(26) $$\ddot{\theta }=\frac{\tau -h\left(\theta ,\dot{\theta }\right)-G\left(\theta \right)}{D\left(\theta \right)}$$

Thus,

(27) $$\ddot{\theta }=D^{-1}\tau +{\ddot{\theta }}_{\nu }+{\ddot{\theta }}_{g}$$

where ${\ddot{\theta }}_{\nu }=-D^{-1}h,\;{\ddot{\theta }}_{g}=-D^{-1}G$.

It is evinced from the above that the acceleration of the moving platform is affected by many factors. However, considering the low velocity of the rehabilitation exoskeleton robot in the working process and the light weight of its own materials, the velocity and gravity factors in the equation are ignored, and the following mapping relationship between the generalized acceleration and driving force is obtained:

(28) $$\ddot{\theta }=D^{-1}\tau$$

All the driving forces applied to the hip joint rehabilitation robot configuration are of unit size, and the direction of action in the joint space is arbitrary. Based on this, all the driving forces are expressed in space and a generalized inertia ellipsoid is formed, which is the dynamic manipulability ellipsoid (DME). The dynamic manipulability ellipsoid can describe the overall performance of the mechanism during motion, including its stability and efficiency. The expression of the ellipsoid is:

(29) $$\tau^{T}\tau =1$$

It can be obtained from Equations (27) and (28):

(30) $${\ddot{\theta }}^{T}{\left(D^{-1}\right)}^{T}\left(D^{-1}\right)\ddot{\theta }=1$$

According to Equation (30), the ellipsoid shape can be used to evaluate the dynamic performance at any point in the workspace. The closer the ellipsoid shape is to the sphere, the better the dynamic performance will be. These points at which the generalized inertia ellipsoid assume completely the form of a sphere become the kinetic isotropic points. At the kinetic isotropic points, the column vectors of the inertial matrix ***D*(*θ*)** are linearly independent of each other and have equal modules. The inertial matrix is a symmetric matrix, that is, the transposed matrix of the inertial matrix is equal to its own inverse matrix. This means that the object can maintain its own motion state during the process of motion, indicating that the object has constant inertia. The inertial matrix and dynamic manipulability ellipsoid play an important role in mechanical dynamics.

The operability of mass matrix ***D*** is related to the ellipsoid formed by the acceleration of the moving platform and the driving force of the joint, which can be expressed as follows:

(31) $$w_{D}=\sqrt{det\left(DD^{T}\right)}=\sigma_{1}\sigma_{2}\sigma_{3}\cdots \sigma_{m}$$

where $\sigma_{1}\geq \sigma_{2}\geq \sigma_{3}\geq \cdots \geq \sigma_{m}\geq 0$.

***k****_D_* is taken as the conditional number of mass matrix ***D***, which is related to the ellipsoidal long axis and short axis of the acceleration of the moving platform, and is used to express the transmission ability of the hip joint rehabilitation robot configuration in any direction, which is expressed as follows:

(32) $$k_{D}=cond\left(D\right)=\frac{\sigma_{\max}\left(D\right)}{\sigma_{\min}\left(D\right)}$$

where $\sigma_{\min}\left(D\right),\;\sigma_{\max}\left(D\right)$ are the least and most singular values of the mass matrix ***D***, respectively.

The dynamic operating degree coefficient (DMI) can be obtained as a local quantitative index of dynamic performance through the above indexes. Its expression is as follows:

(33) $$w=\left|det\left(D^{-1}\right)\right|$$

Then, based on Equation (32), the global dynamic operating degree coefficient (GDMI) can be obtained to describe the overall dynamic performance of the robot. Its expression is:

(34) $$w_{G}=\frac{\int_{w}wdw}{\int_{w}dw}$$

Through the established dynamic performance model, the link lengths of *l*_1_ and *l*_7_ were selected as variables, the two sets of data with a difference of 15 mm were given, and the other link parameters were adjusted along with the overall mechanism to conduct dynamic performance simulation analysis. The workspace of −90°~90° and the workspace of the hip joint motion range were studied. The motion range of the hip joint is shown in Table 4. The cloud image of the simulation results is shown.

**Figure 9:**
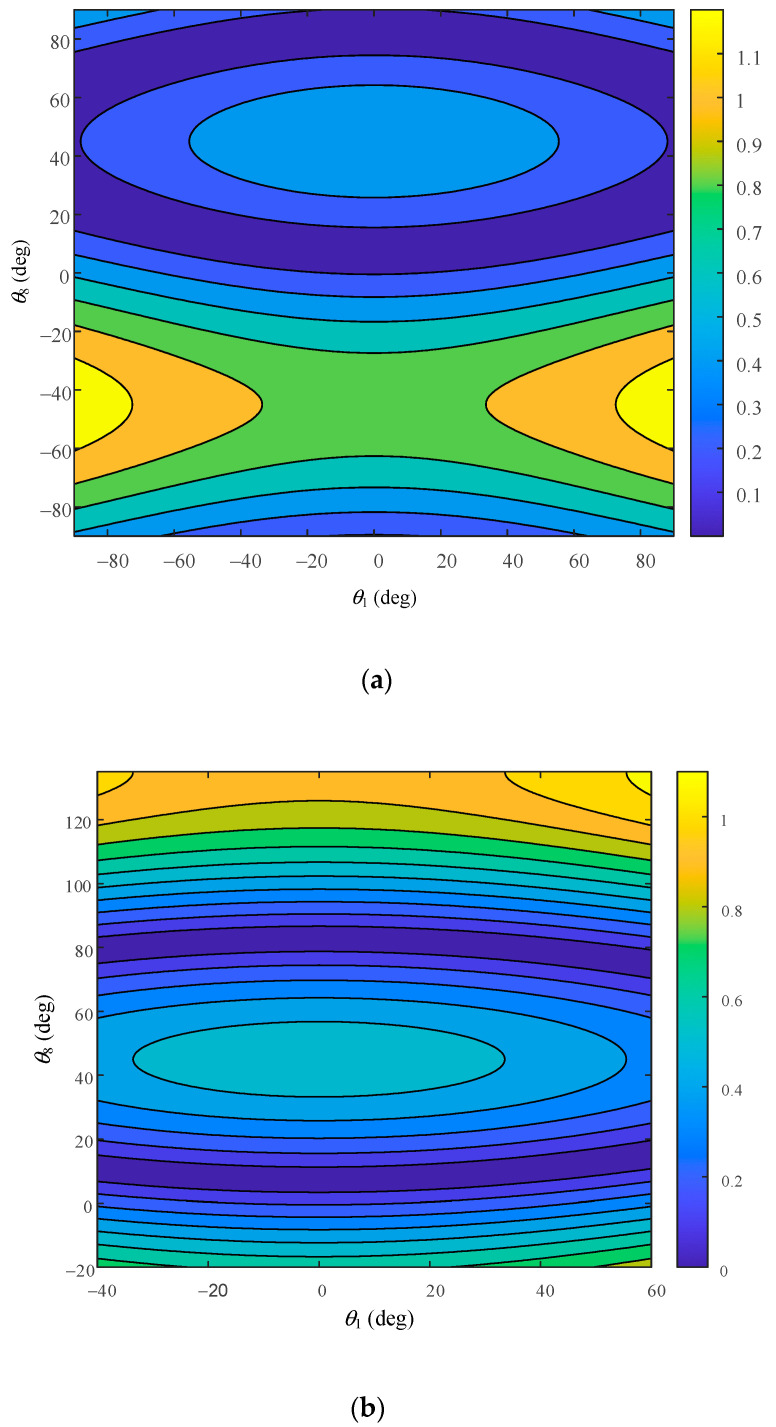
Dynamic performance of hip joint under parameter 1. (**a**) Distribution in the workspace of −90°~90° under parameter 1; (**b**) distribution of hip joint motion range under parameter 1.

Figure 9 shows the ellipsoidal distribution of dynamic performance obtained under the first set of link length parameters. Figure 9a shows its distribution in the range of the −90°~90° workspace. Based on the distribution of Figure 9a and the range of motion of the hip joint, Figure 9b is drawn. It can be seen that the dynamic operating degree coefficient is centrally distributed between 0.5 and 0.7.

**Figure 10:**
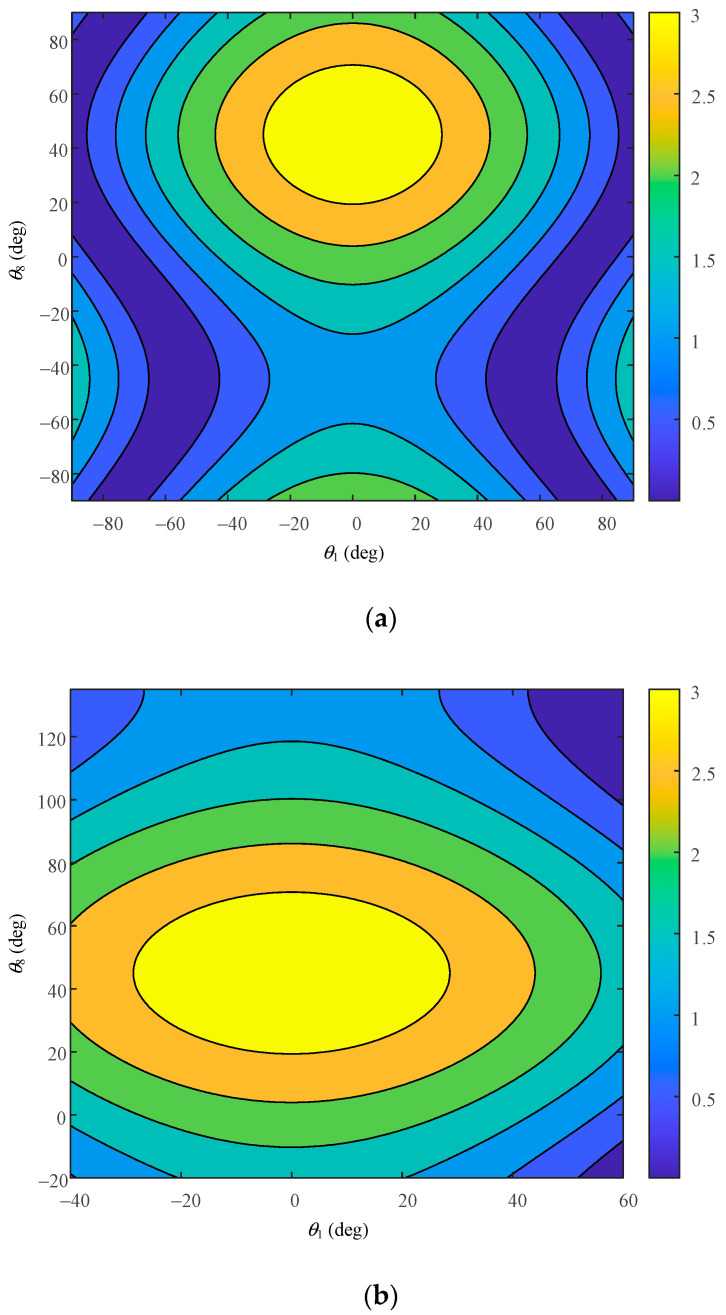
Dynamic performance atlas of hip joint under parameter 2. (**a**) Distribution atlas in the working space of −90°~90° under parameter 2; (**b**) distribution atlas in the working space of −90°~90° under parameter 2.

According to the second set of link length parameters, the ellipsoid distribution of the dynamic operating degree is obtained, as shown in Figure 10. Figure 10a still shows the ellipsoid distribution in the range of the −90°~90° workspace, and its values are centrally distributed between 0.8 and 1.3. Figure 10b shows the ellipsoidal distribution atlas of the dynamic operating degree calculated according to the motion range of the hip joint, which is mainly distributed between 1.3 and 2.5. This parameter is obviously not applicable to this mechanism, and its dynamic performance is poor, therefore failing to meet the requirements for a good performance of the mechanism.

In summary, by comparing the ellipsoidal distributions of the two groups of link length parameters, it is found that the obtained ellipsoidal distributions of the dynamic performance operability are symmetrically distributed, a fact which is in accordance with the overall characteristics of the robot configuration. Based on the first set of link length parameters, the dynamic performance indexes are mainly distributed between 0.5 and 0.75, which satisfies the ellipsoidal coefficient of dynamic operability obtained with the equation. The dynamic performance based on the second set of link length parameters is obviously poor, and it does not satisfy the link relation expression. The first set of link length parameters is the original design parameters, indicating that it has a good dynamic performance.

## 5. Conclusions

A hip joint rehabilitation robot configuration is analyzed, and its velocity and acceleration during forward flexion, extension, and abduction/adduction movements are analyzed. The velocity and acceleration curves for these movement types are given, and its great movement performance is verified. The Newton–Euler method is used to build the dynamic model. Based on the dynamic model, the change curves of force and moment under different motion types are given, and the reasonable changes in force and moment are analyzed. Finally, combined with the dynamic model, the dynamic performance index of the robot is obtained, and the dynamic performance of the robot under different link length parameters of *l*_1_ and *l*_7_ is determined. By comparing it with the initial rod length where *l*_1_ = 47 mm and *l*_7_ = 172 mm, the rationality of the original link length design is obtained. Through the dynamic analysis of the hip joint rehabilitation robot and its dynamic performance, it can be seen that the design of the mechanical structure is reasonable. The simulation analysis results show that the force and moment change law of the mechanism during movement indicate a good dynamic performance. Through the above work, on the basis of obtaining the dynamic model and dynamic performance index of the mechanism, it is evinced that the mechanism has a simple structure and good dynamic performance. According to these results, it can be concluded that the application of the mechanism in the hip joint rehabilitation robot can simplify the control of the robot and increase the comfort of the robot because of the rationality of its mechanical structure. This lays a foundation for follow-up work and provides a theoretical reference for future institutional research in the field of rehabilitation robots.

## Figures and Tables

**Table 1:** Parameters of the 5R branch chain.

*i*	*α_i_*_−1_/(°)	*a_i_*_−1_/mm	*θ_i_*/(°)	*d_i_*/mm
1	0	0	*θ*_1_	0
2	90	0	*θ*_2_	*l*_1_
3	−90	0	*θ*_3_	*l*_2_
4	0	0	*θ*_4_	*l*_3_
5	0	*l*_4_	*θ*_5_	0

**Table 2:** Parameters of the 2R branch chain.

*i*	*α_i_*_−1_/(°)	*a_i_*_−1_/mm	*θ_i_*/(°)	*d_i_*/mm
1	0	0	*θ*_7_	0
2	−90	0	*θ*_8_	*l*_7_

**Table 3:** Symbol glossary.

Notation	Explanation
***T***	Transformation matrix of the D-H matrix.
T	Transpose of a matrix.
***p***	The translation vector of the link relative to the previous link.
***ω***	Representative angular velocity.
ν	Representative velocity.
***R***	Representative of the transformation matrix used in the Newton–Euler method.

**Table 4:** Motion range of the hip joint.

Kinesthetic Learner	Motion Range/(°)
Flexion	0~125
Extension	0~30
Adduction	0~60
Abduction	0~40

## Data Availability

Some of the data supporting this study are in the published papers of the first author of this work, and the data can be found in [Applied Sciences] at [https://doi.org/10.3390/app122312488], reference number [25].
